# Ultrafast Biomarker Quantification through Reagentless Capacitive Kinetics

**DOI:** 10.1021/acs.analchem.2c05398

**Published:** 2023-03-01

**Authors:** Shaoyu Kang, Mohamed Sharafeldin, Sophie C. Patrick, Xuanxiao Chen, Jason J. Davis

**Affiliations:** † Department of Chemistry, University of Oxford, South Parks Road, Oxford, OX1 3QZ, United Kingdom; ‡ Department of Chemistry, University of Otago, Dunedin 9054, New Zealand

## Abstract

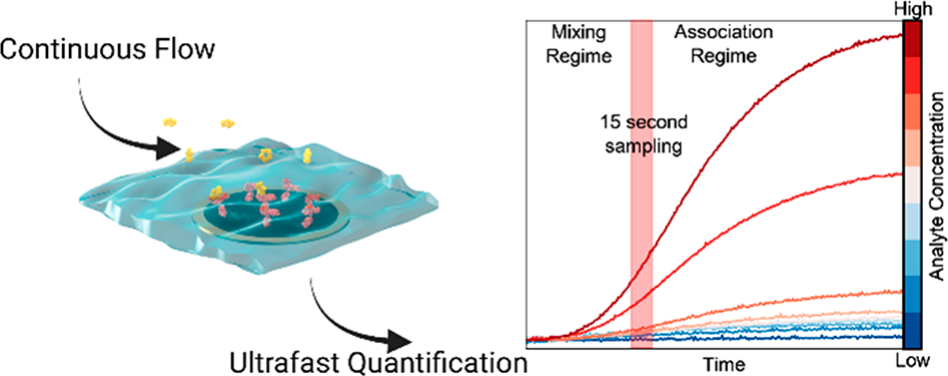

We introduce a facile assessment of binding kinetics at bioreceptive redox-active interfaces as a means of quantifying target proteins. This is achieved by monitoring the redox capacitance (*C*_r_) of a receptor-modified conductive polymer interface under continuous flow. Exemplified with the quantification of C-reactive protein (CRP), capacitance analyses resolve both the association and dissociation regimes in real-time. Significantly, the rate of electrochemical signal change within the association regime is a sensitive function of target concentration, enabling marker assaying down to picomolar levels, comparable to end-point assays, in 15 s. This reagentless proof-of-principle methodology is envisioned to be widely applicable to the facile quantification of a range of other pertinent, clinically relevant targets.

## Introduction

The quantification of protein biomarkers lies central to a range of clinical applications, including the prediction, prognosis and diagnosis of a plethora of disease states.^1^ Despite significant progress in correlating the levels of a growing library of markers to associated clinical conditions, developing a facile, sensitive, and selective quantification method in a scalable format remains challenging. The well-established enzyme-linked immunosorbent assay (ELISA) remains limited as a multistep laboratory-based method with a number of scaling and practical issues.^2^ A broad range of alternative optical and electrochemical methods have been developed for protein biomarker quantification, with the latter particularly well-suited for widespread clinical applications due to its associated high sensitivity, ease of operation, simple hardware requirement, and compatibility with microelectronic device integration.^3^ These electrochemical techniques can be collectively classified into labeled and label-free formats, where the former typically utilizes a probe-carrying (enzyme, redox-active molecule, or nanoparticle) secondary antibody, and the latter monitors the electrochemical perturbation (typically voltammetric or impedimetric) of an interface upon the selective recruitment of targets. The vast majority of such analyses are end-point assays, wherein analytes are incubated at the modified interfaces for an optimized period of time and the associated change in electrochemical signal used to assess target concentration. This format is typified by extended incubation times (typically >15 min),^4^ and additionally requires either an enzyme substrate incubation or target labeling. Such characteristics impede practical translation, particularly in point-of-care settings with patients who are rapidly deteriorating or are highly infectious.^3,5^

An assessment of the binding kinetics associated with an interfacial recognition event has been shown to enable the quantification of target analytes by surface plasmon resonance (SPR),^6^ biolayer interferometry (BLI),^7^ or field-effect transistors (FET).^8^ These prior works, though, require both expensive hardware/specific microfabrication and a detailed statistical analysis of full (end point) sensograms prior to quantification. Reagentless electrochemical assays are easily integrated within microfluidic platforms and can afford a fast, sensitive, and highly practical approach for evaluating binding kinetics.^9^

Electrochemical capacitance spectroscopy (ECS) can be utilized to probe recognition at an appropriate redox-active receptive interface. This binding-dependent capacitive fingerprint supports sensitive reagentless sensing^10−12^ in a manner that can be readily integrated into a microfluidic continuous-flow format.^13^ Previously, we have shown that antibody-decorated redox-active electropolymerized polyaniline (PANI) films respond sensitively to target recruitment.^12^ Herein, we examine the use of real-time redox capacitance signals, acquired over just 15 s, to determine binding association kinetics and to assay specific targets. This is an experimentally simple, scaleable, and reagentless method that supports an unprecedented speed of target quantification.

Prior to examining the temporal data acquired under continuous flow, we first provide a brief overview of the kinetics that underpin interfacial binding processes more generally. The target binding rate at an appropriate receptive interface is governed by (1) mass transport of the target from the bulk solution and (2) the subsequent specific molecular binding event; these have associated mass transfer, *k*_mt_, and association rate constants, *k*_on_, respectively.^14−16^ There are, based on the relative magnitudes of these rates, three distinct regimes that can be identified; namely, those of the mass transport limited (MTL), partial MTL, and a binding-limited regime. Under continuous flow in a microfluidic setup, the analyte flux to the receptive interface depends on the associated fluidic dimensions, flow rate (*u*), molecular diffusion coefficient (*D*), and the bulk analyte concentration (*c*_analyte_), as indicated by eq 1; here *J* is the flux of analyte, with *w*, *l*, and *h* representing the width, length, and height of the fluidic compartments, respectively.^17,18^
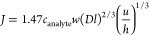
1

The MTL regime is limited by analyte diffusion,^16,17,19,20^ where, for a typical protein, *k*_mt_ is in the range of 10^8^ M^–1^·sec^–1^.^19^ Within this regime, the surface concentration of the analyte (*c*_surface_) is diffusion limited and proportional to the square root of the incubation time (, eq 2),^21^ following Fick’s diffusion law.
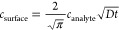
2

In the binding-limited regime, interfacial recruitment kinetics are limited by molecular binding, most normally when *k*_on_ is less than 10^5^ M^–1^·sec^–1^.^22^ One expects the temporal response here to be exponential in nature until equilibrium is established, i.e., a pseudo-first-order kinetic model applies (eq 3), where *f*_b_ is the fraction of bound target, defined relative to the total number of available binding sites, and *f*_b,eq_ is the fraction bound at equilibrium. *k*_obs_ is the observed rate constant defined as *c*_analyte_ × *k*_on_ + *k*_off_.
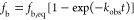
3

Outside of these two limits, molecular recognition at immunoaffinity interfaces with a *k*_on_ > 10^5^ M^–1^·sec^–1^ is most typically observed to take place in a partial MTL regime;^15,23^ even when affinities are high, biomolecular binding typically involves some degree of conformational rearrangement;^19,24^ this can then make the overall binding rate comparable to the mass transport rate.^15^

Within the MTL regime, this is given by eq 2 above. Within a binding-limited regime, the net response (*v*_overall_) is governed by the relative magnitudes of the association (*v*_on_) and dissociation rates (*v*_off_), eq 4.^14,20^ When surface ligand concentrations (*c*_ligand_; most typically ∼10^–12^ mol·cm^–2^)^25^ are in vast excess of the surface analyte concentration, binding is limited by *c*_analyte_. Initially ligand occupancy is close to zero, analyte dissociation negligible, and the temporal response depends on bulk analyte concentration (*c*_analyte_) only (eq 5).
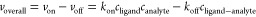
4
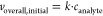
5

**Figure 1:**
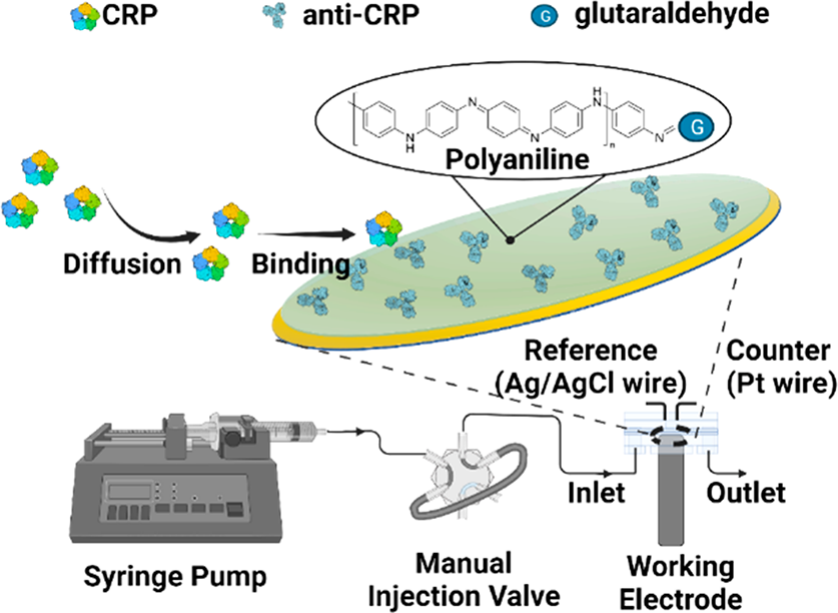
Schematic depiction of the continuous flow microfluidic configuration housing an anti-CRP/PANI modified conventional Au disc electrode for electrochemical capacitance spectroscopy (ECS) assays. The electrode is inserted into a custom 3D printed microfluidic cell with a Ag/AgCl wire reference electrode and Pt wire counter electrode. The sample and running buffer are delivered at a controlled flow rate by an automated syringe pump. The flow rate is accessible up to 500 μL·min^–1^. Temporal resolution is a function of the ECS AC frequency (∼2 s).

In all three regimes, the temporal response at the recruiting interface is analyte concentration-dependent. We hypothesized here, then, that a sensitive monitoring of electrochemical capacitance, and, specifically, the rate at which it changes within this initial regime (across a few seconds) would thus support an analyte quantification. This high-resolution temporal analysis was performed in real-time using 3D-printed microfluidic chips housing a conventional, appropriately modified, disc electrode (Figure 1). Nonspecific adsorption was minimized by standard surface blocking procedures, and all responses were normalized to the re-established baseline prior to each injection to negate any contribution from interferants (see the Experimental Section of the main text for details of the response normalization).

## Experimental Section

### Materials and Instruments

Information concerning the materials and instruments used throughout is given in the Supporting Information.

### Bioreceptive Interface Preparation

The anti-CRP/PANI interface was prepared following our previously reported protocol.^12^ Briefly, 1 mL of 98% aniline was mixed with 2 mL of 50% phytic acid, followed by addition of 17 mL of deionized water. Electrodeposition of PANI was performed by submerging Au disc electrodes in the prepared aniline solution and running chronopotentiometric scan at a current density of 10 μA·cm^–2^ for 10 min to control the thickness of the generated PANI films. The PANI-functionalized electrodes were then washed with deionized water, incubated in a solution of 2.5% glutaraldehyde (in water) for 30 min at room temperature, then rinsed with 0.1 M PB buffer. Antibodies were immobilized onto the surface by incubating these electrodes with 100 μg·mL^–1^ polyclonal anti-CRP solution for 18 h. Finally, the anti-CRP/PANI electrodes were rinsed with PB buffer, then blocked using ThermoFisher SuperBlock (TBS) blocking buffer for 30 min to reduce nonspecific binding. Electrodes were then washed and kept in PB buffer before measurements.

### End-Point CRP Assay

Changes in *C*_r_ were assessed by shifts in the inflection point of capacitive Nyquist plots (Figure S3, SI), performed at fixed potential, *E* (*E* = half-wave potential, *E*_1/2_ of the PANI/PANI^+^ couple = −0.16 V vs Ag/AgCl) over a frequency range of 0.1–100000 Hz. The ECS baseline stability of the anti-CRP/PANI interface was evaluated by running 3 blank measurements after incubating the electrodes in PB buffer for 20 min. Electrodes were then incubated for 10 min with 50 μL of increasing concentrations of recombinant CRP (from 40.0 ng·mL^–1^ to 10.0 μg·mL^–1^). After incubation with each concentration, electrodes were washed 3 times with PB buffer, and the shift in *C*_r_ was determined from the average of the inflection points from three repeat Nyquist plots. *C*_r_ is reported as the relative response (RR) according to the equation RR/% = (1 – *C*_r,t_)/*C*_r,0_ × 100, where *C*_r,t_ = redox capacitance after incubation, *t*, and *C*_r,0_ = redox capacitance in blank solution, and fitted to the Langmuir–Freundlich isotherm to determine the binding constant, *K*_a_.

### Continuous Flow Assays

Continuous redox capacitance, *C*_r_ measurements were performed at fixed potential, *E* (*E* = half-wave potential, *E*_1/2_ of the PANI/PANI^+^ couple = −0.16 V vs Ag/AgCl) and fixed frequency, *f* (*f* = *f*_r_, AC frequency determined from the inflection point of the capacitive Nyquist plot). *C*_r_ was monitored in real-time in conjunction with a microfluidic system with a temporal resolution of 2 s, whereby PB buffer was continuously pumped over the interface at a flow rate of 25 μL·min^–1^. Once a stable baseline was established, increasing CRP concentrations from 40.0 ng·mL^–1^ up to 10.0 μg·mL^–1^ were injected sequentially. Each injection was followed by a washing step with PB buffer over a 20 min window to re-establish the baseline prior to the next injection (CRP binding is partially reversible, see later for further discussion). Specificity was assessed from the *C*_r_ response to injecting 1.0 mg·mL^–1^ HSA, IgG, and BSA in an identical manner to that above. Percentage recoveries were calculated from comparison of the *C*_r_ responses of the anti-CRP/PANI interfaces to 2.50, 5.00, and 10.0 μg·mL^–1^ CRP samples, with or without 1.0 mg·mL^–1^ BSA or 1.0% human serum.

### Data Analysis

All data analysis was performed with Origin2021 or MATLAB software. The raw redox capacitance data acquired in real-time was converted to a relative response according to the equation RR/% = (1 – *C*_r,t_)/*C*_r,0_ × 100 (where *C*_r,t_ = redox capacitance at a given time, *t*, and *C*_r,0_ = initial redox capacitance at *t* = 0). Data was normalized to each re-established baseline prior to each injection of CRP. The observed binding rate constant, *k*_obs_ was obtained from fitting relative *C*_r_ responses to eq 3. By plotting *k*_obs_ versus analyte concentration *c*_analyte_, the association, *k*_on_ and dissociation, *k*_off_ rate constants can be obtained from the slope and intercept, respectively. *c*_analyte_ was quantified according to eq 5, from the slope of the *C*_r_ signal over a 15 s window.

## Results and Discussion

### Responsive Anti-CRP/PANI Film Characterization

**Figure 2:**
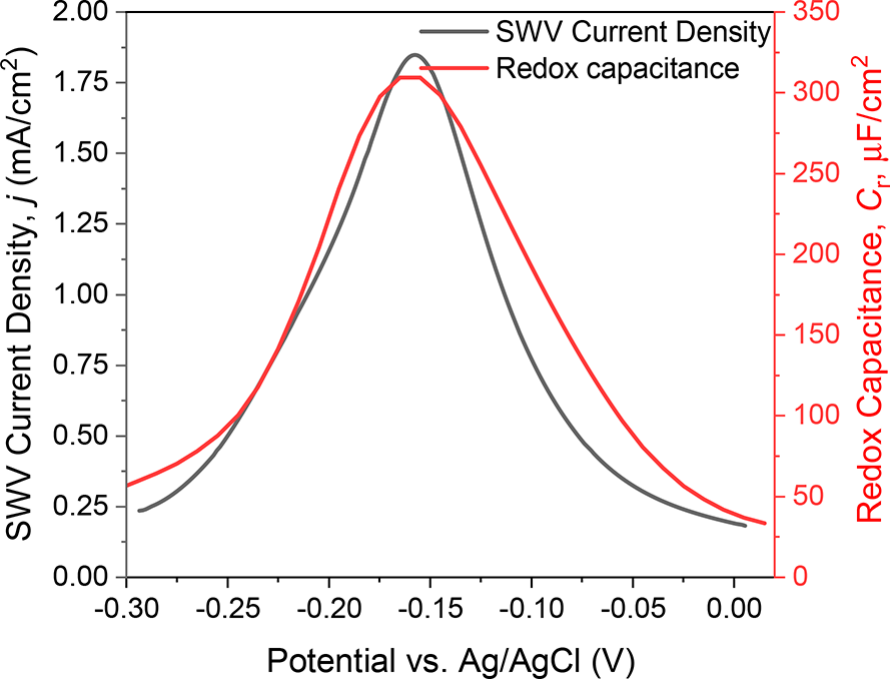
Representative PANI film redox capacitance (red) and SWV (black) fingerprints. The AC frequency of redox capacitance scan (red) was selected from the inflection point of capacitive Nyquist plots (Figure S3, see SI).

Continuous-flow electrochemical capacitance spectroscopy was performed on antibody-modified PANI interfaces (Figure 1).^12^ PANI was first electropolymerized onto gold disc electrodes via chronopotentiometry at a current density of 10 μA·cm^–2^ for 10 min. The resultant film’s FT-IR (Figure S1, SI) and contact angle (43.7°) features were consistent with expectations.^26^ AFM images demonstrated that the generated films were 6.4 ± 1.1 nm in thickness (average surface roughness *R*_a_ = 1.6 ± 0.12 nm; Figure S2, SI). A distinct redox peak corresponding to the PANI/PANI^+^ half-wave potential is observed at −0.16 V (vs Ag/AgCl), as assessed by both capacitance spectroscopy (potential scanning at a fixed frequency, see SI for more detail),^27^ and square wave voltammetry (SWV) in Figure 2.^12^

Anti-CRP was covalently integrated by glutaraldehyde cross-linking, which was associated with an increase in water contact angle from 43.7° (PANI)^12^ to 56.3° (anti-CRP/PANI) and a decrease in redox capacitance *C*_r_ at the same half-wave potential (as assessed from the inflection points of the capacitive Nyquist plots in Figure S3, and *C*_r_ peak in potential scan shown in Figure S4), consistent with expectations.^28^ SPR analysis of anti-CRP/PANI interface indicated high levels of antibody integration (192.9 ± 9.0 ng·cm^–2^), as estimated using the standard Reichert protocol,^29^ corresponding to a surface coverage of 60.8 ± 2.8% of a theoretical monolayer of IgG antibodies.^30^

### Static (End-Point) CRP Assays

Prior to assessing the transduction of specific target recognition, the ECS signal baseline stability was analyzed in buffered solution where less than 1% (<2.0 μF·cm^–2^) drift over a 10 min measurement period was typically observed (Figure S5, SI) and <5% across 3–4 h of continuous measurement. Anti-CRP modified electrodes exhibited a concentration-dependent *C*_*r*_ response to target recognition in subsequent end-point assays (see Experimental Section), empirically fitted with a Langmuir–Freundlich isotherm (Figure S6, SI); the associated linear dynamic range spanning from 80.0 ng·mL^–1^ (651 pM) up to 10.0 μg·mL^–1^ (87.7 nM) with a picomolar limit of detection (LOD = 3σ/*s*, 58.1 ± 6.2 pM) (Figure S7, SI).^12^

### Resolved Target Binding Kinetics

**Figure 3:**
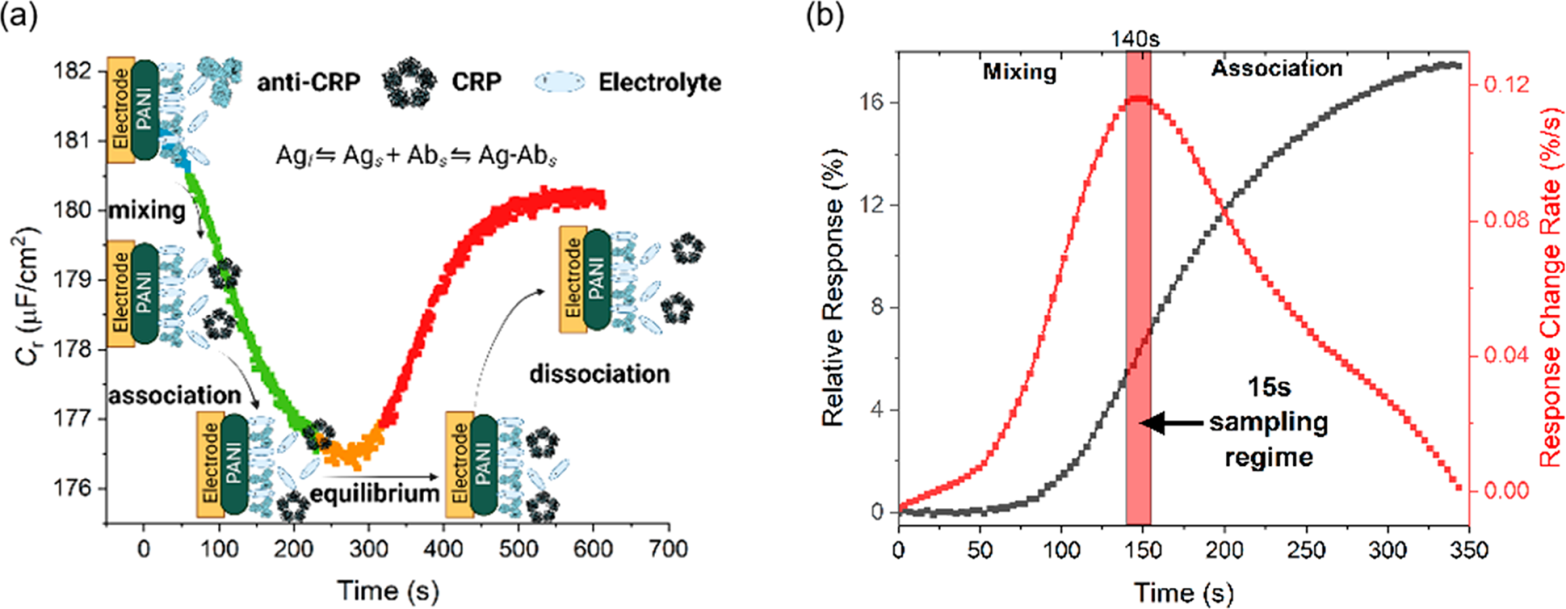
(a) Representative redox capacitance (*C*_r_) measurement showing changes associated with antigen recognition under flow (2.50 μg·mL^–1^ CRP injection at a flow rate of 25 μL·min^–1^), with the mixing (blue), association (green), equilibrium (orange), and dissociation stages (red) resolved. The subscripts of antibody (Ab) and antigen (Ag) indicate their states, where “*l*” represents bulk solution and “*s*” represents the interface. (b) The relative response (black) of *C*_r_ and the rate of response change (red) in response to a CRP injection underflow. The response change rate is directly calculated from the first derivative of capacitive relative response, presenting the CRP overall binding rate (*v*_overall_). Mixing and association stages are separated by the red rectangle, with the sampling regime indicated after the 140 s mixing stage. All data were directly analyzed from 2.50 μg·mL^–1^ CRP injection underflow at a flow rate of 25 μL·min^–1^ (further details of this continuous flow assay can be found in the Experimental Section of the main paper, and a full sensogram is shown in Figure S9).

Real-time continuous flow immunoassays were performed using a custom 3D printed microfluidic cell (cell internal volume *V*_cell_ ∼ 60 μL, see more detail in Figure S8, SI), connected to a manual sample injection valve with a sample loop volume of 100 μL. Temporal *C*_*r*_ analyses were monitored at the frequency of inflection from capacitive Nyquist analyses (see Figure S6, SI). Both association and dissociation regimes are clearly resolved (Figure 3 a), within four regimes, namely those of mixing, association, equilibrium and dissociation, being presented. The duration of the mixing stage was estimated based on the time required to displace the buffer (inside the microfluidic cell) with the injected sample. This also determines the starting point of the association regime, where (eq 4) the association rate is predicted to be at its maximum. The data here (i.e., the sampling window shown in Figure 3b) is almost exclusively association only. At a 25 μL·min^–1^ flow rate, the time required for the buffer displacement is ∼140 s (this is flow rate-dependent, falling with faster flow), as depicted in the derivative relative response shown in Figure 3b. The small apparent decrease in *C*_r_ observed during the mixing stage (Figure 3a) is attributed to buffer displacement and some interfacial association.^31^ Following mixing, *C*_r_ signals report on target association (Figure 3a)^11^ until the interface equilibrates and a plateau is reached (see eq S1 and Table S1, SI). In this regime, the response first derivative (red curve, Figure 3b) is a constant. This entire window of events is, of course, largely (∼90%) reversible with CRP dissociation under continuous flow (Figure 3a).

To confirm operation in the partial MTL regime,^14^ we first estimated the apparent net rate constant (*k*_obs_ = *k*_on_·*c*_analyte_ + *k*_off_) by fitting *C*_r_ responses to eq 3; this afforded an association rate constant of *k*_on_ = 1.19 (±0.04) × 10^5^ M^–1^·s^–1^ (Figures S10 and S11, SI) in excellent agreement with SPR analyses at the same interface (*k*_on_ = 1.16 (±0.15) × 10^5^ M^–1^·s^–1^). Although higher flow rates accelerate association (eq 1 and Figure S12, SI), binding remains in the partial MTL regime across all accessible flow rates (1.0–500 μL·min^–1^). This is supported by a lack of linearity in the response versus  (eq 2). Binding is pseudo-first-order (eq 3) at all accessed fixed flow rates;^15^ the binding rate decays exponentially with time (see the first derivative of the capacitive relative response after the mixing regime, (Figure 3b) and is predictably maximal during the sampling interval, as expected when binding kinetics are contributing significantly to interfacial signal growth.^15^

### CRP Quantification Under Continuous Flow

**Figure 4:**
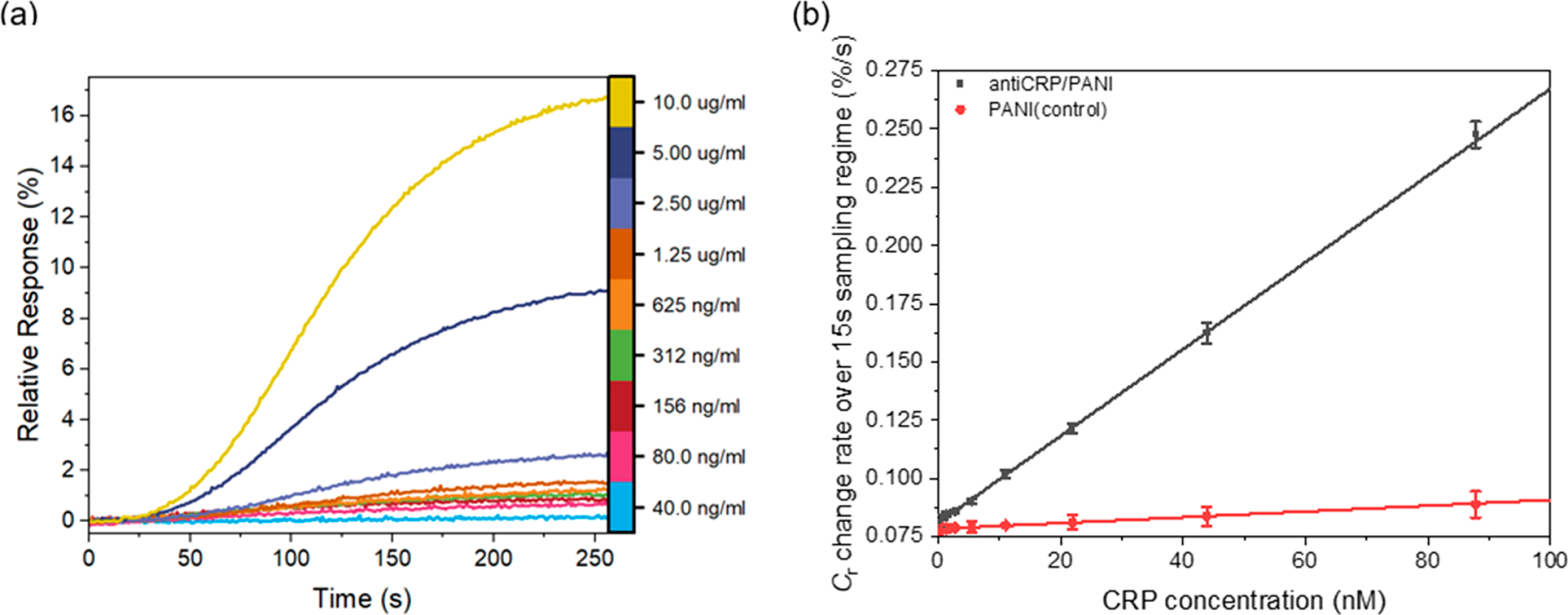
(a) Representative sensograms of a continuous-flow ECS response at various concentrations of CRP from 40.0 ng·mL^–1^ (343 pM) to 10.0 μg·mL^–1^ (87.7 nM) with a flow rate of 25 μL·min^–1^ (a full sensogram is shown in Figure S9, further details of the continuous flow assays can be found in the Experimental Section of the main paper). (b) Slope of *C*_r_ response during the first 15 s of the association regime at an anti-CRP modified PANI interface (black) and PANI electrode (control, red) to increasing concentrations of CRP. Error bars represent one standard deviation of three individual electrodes (*n* = 3), errors are typically <5%.

After confirmation of pseudo-first-order kinetic conditions (and a linearized relationship between bulk analyte concentration and response rate), continuous flow assays were performed to analyze the association regime (Figure 4a) as a function of analyte concentration (*C*_r_ response to targets was shown in Figure S9, SI). We can specifically note that the initial (prior to any contribution to signal from dissociation; see Figure 3b) rate of change of *C*_r_ (after mixing) is proportional to CRP bulk concentration (eq 5 and eqs S2–S6). Ideally, the sampling window should be in a regime where the first derivative of the response is maximal and stable, with no significant contribution from dissociation (plateau in the first derivative curve of Figure 3b). An evaluation of linear fitting error indicated that a 15 s sampling in this window had a relative quantification error <5% (see eqs S7–S13; the relative error of linear fits across different sampling windows is shown in Figure S14 and Table S2). This 15 s analysis window supports the generation of “dose–response” curves (Figure 4b) with a dynamic range spanning 80.0 ng·mL^–1^ (651 pM) to 10.0 μg·mL^–1^ (87.7 nM; Figure 4b), and an associated LOD of 70 pM, comparable to that of the end-point assay (see Figure S6, SI; but over 15 s rather than tens of minutes). These analyses demonstrated excellent reproducibility with standard deviations (std) <3% across three individual measurements (on the same working electrode) and <5% across three different electrodes.

**Figure 5:**
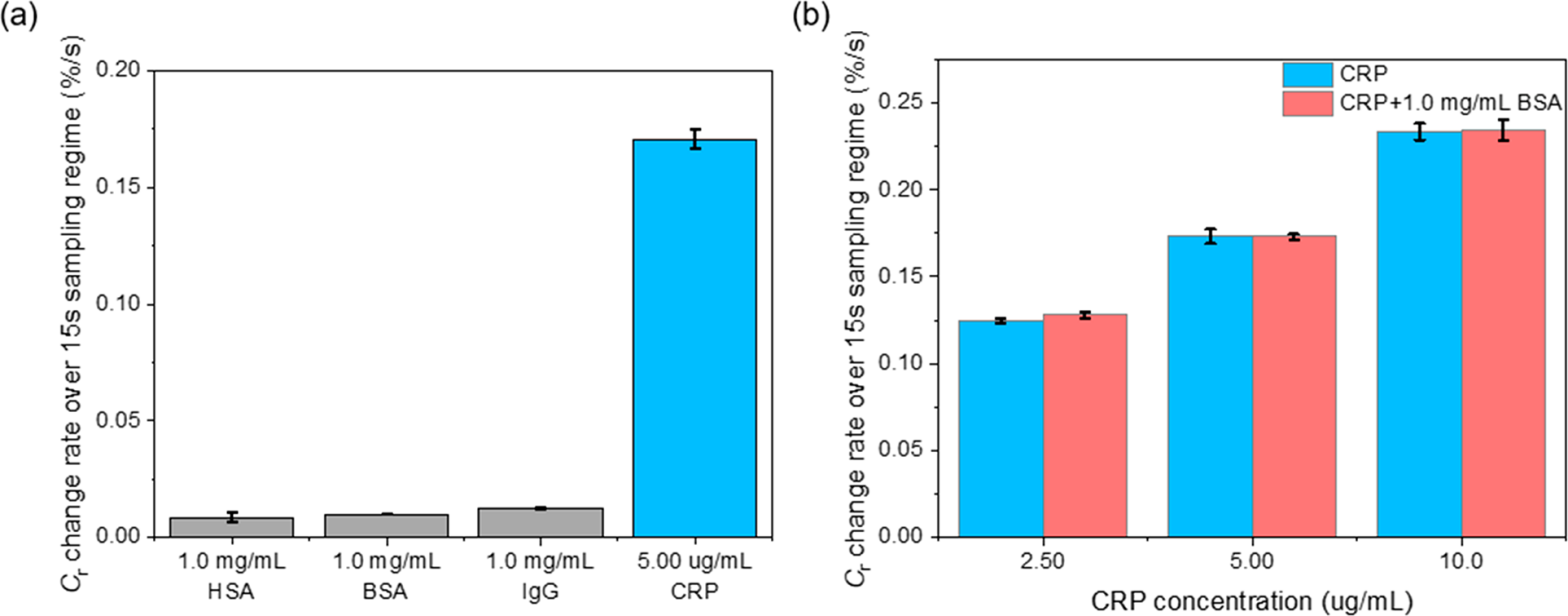
(a) Rate of *C*_r_ change over first the 15 s of anti-CRP/PANI interface toward 1.0 mg·mL^–1^ HSA, BSA, and IgG, and 5.00 μg·mL^–1^ CRP underflow with a flow rate of 25 μL·min^–1^. (b) The recovery of 2.50, 5.00, and 10.0 μg·mL^–1^ CRP in 1.0 mg·mL^–1^ BSA matrix over anti-CRP/PANI interface with continuous-flow ECS measurement. Error bars represent the standard deviations of three individual measurements each concentration (*n* = 3).

Interfacial specificity was evaluated by injecting 1.0 mg·mL^–1^ of human serum albumin (HSA), bovine serum albumin (BSA), and bovine immunoglobulin G (IgG) under the same flow conditions. As shown in Figure 5a, the slopes of the 15-s sampling window are generally <5% compared to that obtained after 5.00 μg·mL^–1^ CRP injection. Herein, the calculated concentrations of CRP spiked BSA solutions showed recoveries of 100.9 ± 1.1% (Figure 5b). In addition, the recovery of CRP (spiked in 1.0% human serum) was quantified at 108.9 ± 2.2% in excellent agreement with independent SPR measurements from the same samples (these giving recoveries of 103.6 ± 4.6%, see Figure S13, SI).

The results above demonstrate a promising approach for assessing Ag-Ab binding kinetics at electrochemical interfaces using a label-free reagentless electrochemical platform. The sensitivity of the PANI-based interface herein is comparable to that of analogous CRP biosensors based on amplified electrochemical, ELISA or electrochemiluminescence (ECL) formats,^32^ all of which are markedly more complex and time-consuming. Temporal resolution is high (∼2 s) and enables target binding to be monitored in real-time. We have specifically demonstrated that just 15 s of this (where dissociative contributions are negligible) can support a statistically robust and sensitive (clinically relevant)^33^ quantification. The interfaces are simple, readily prepared, and readily scaleable.

## Conclusion

This work supports the use of the temporal change in redox capacitance to sensitively quantify target analyte concentration. The analyses, spanning 15 s, are reagentless, label-free, and utilize the native capacitive fingerprint of an antibody-supporting electropolymerized film as the signal generator. Measurements utilize a single-step immunorecognition event and are readily integrated within a simple and scalable 3D-printed microfluidic platform. We believe this represents an entirely new marker quantification tool and is readily applicable to any marker for which a high affinity receptor exists.

## Supporting Information Available

Additional experimental details, materials, instruments, and relevant protocols. This includes characterization of the interface, the microfluidic configuration, immunoassay results obtained by SPR, electrochemical methods, and derivation of relevant equations (PDF)
