# Supplementary material for: Ultrafast Biomarker Quantification through Reagentless Capacitive Kinetics
Source: Anal Chem. 2023 Mar 1;95(10):4721–7. doi: 10.1021/acs.analchem.2c05398 (PMC10018454; doi:10.1021/acs.analchem.2c05398)
Supplement: Supplementary file 1 — ac2c05398_si_001.pdf [file ac2c05398_si_001.pdf]

SUPPORTING INFORMATION

Ultrafast biomarker quantification through reagentless capacitive kinetics

Shaoyu Kang,§ Mohamed Sharafeldin,§† Sophie C. Patrick,§ Xuanxiao Chen,§ Jason J. Davis*§

* Corresponding author

§ Department of Chemistry, University of Oxford, South Parks Road, Oxford, OX1 3QZ, U.K.

† Department of Chemistry, University of Otago, Dunedin 9054, New Zealand

Table of Contents

Experimental Section S-1-S-3

Figure S1. FT-IR spectrum of PANI film S-4

Figure S2. AFM images and profiles of PANI film S-4

Figure S3. Capacitive Nyquist plots during interface preparation S-5

Figure S4. Redox capacitance of PANI and anti-CRP/PANI interfaces as a function of potential S-5

Figure S5. Interfacial stability of anti-CRP/PANI film S-6

Figure S6. Capacitive end-point CRP immunoassay under static conditions S-6

Figure S7. Linear fitting of results from capacitive end-point CRP immunoassay S7

Figure S8. Schematic of microfluidic setup S-7

Figure S9. Continuous ECS sensogram of CRP immunoassay S-8

Figure S10. SPR sensogram of CRP immunoassay S-8

Figure S11. Binding rate constant vs CRP concentration plots obtained from SPR and continuous flow ECS data S-9

Figure S12. Influence of flow rate on CRP binding S-9

Figure S13. Recovery of anti-CRP/PANI interface in 1.0 % human serum S-10

Equation S1. Derivation of association binding kinetics model S-11

Table S1. Estimation of time required for CRP binding to reach equilibrium S-11

Equation S2-S13. Proof of linear approximation of the association regime and its associated errors S-12-S-13

Figure S14. Experimental errors associated with sampling window duration S-14

Table S2. Estimated errors associated with sampling window duration S-14

References S-14

EXPERIMENTAL SECTION

Materials and Instruments

Sodium phosphate dibasic, sodium phosphate monobasic, phytic acid solution (50% w/w in H_2O), aniline, glutaraldehyde solution (25% in water), albumin from human serum (HSA), IgG (from bovine serum, ≥ 95%), and bovine serum albumin (BSA) were purchased from Sigma (UK). Native human C-Reactive protein (CRP) and Goat anti-CRP were purchased from Bio-Rad Laboratories, Inc. (UK). SuperBlockTM (TBS) blocking buffer was purchased from ThermoFisher Scientific (UK). All chemicals were used without further purification.

Fourier transform infrared spectra (FT-IR) were obtained from an FT-IR spectrophotometer (IRTracer-100, Shimadzu, Japan). Surface plasmon resonance (SPR) was performed with a Biacore SPR instrument (Biacore X100, Cytiva). Water contact angles were analyzed with a FTA1000B, First Ten Angstroms, Inc. goniometer. Atomic force microscopy (AFM) images were obtained with a NanoWizard 3, JPK Instruments atomic force microscope. The continuous flow set-up is comprised of a custom, 3D-printed microfluidic chip (cell volume of around 60 μL, obtained with an ELEGOO Mars UV photocuring 3D printer, ELEGOO, Inc.), an injector (Rheodyne 9725, with a sample loop volume of 100 μL), a syringe pump (PHD ULTRA Syringe Pump, Harvard Apparatus, UK) and a 3-electrode electrochemical cell (see below).

All electrochemical experiments were performed using a PalmSens potentiostat (PalmSens BV) with a three-electrode system, consisting of a gold (Au) working electrode (1.6 mm electrode diameter, BASi), a platinum wire (Pt) counter electrode, and a silver/silver chloride wire (Ag/AgCl) pseudo reference electrode. Electrochemical measurements were conducted in 0.1 M phosphate buffer (PB) buffer with a pH of 7.4, unless otherwise stated. Electrochemical impedance spectroscopy (EIS) was conducted over a range of 40 frequencies from 100 kHz to 0.1 Hz with a sigmoidal AC perturbation of 5 mV, and the DC potential was fixed at the half-wave potential of polyaniline (- 0.16 V, vs. Ag/AgCl). The capacitance of the interface was obtained according to $C'=Z''/Z^2$ and $C''=Z'/Z^2$, where ω is the angular frequencies and Z is the impedance of the interface.[1] C_r was obtained from the diameter of the semicircle or inflection point in the capacitive Nyquist plot. Density of states measurements (i.e. redox capacitance vs. potential) were performed over a potential range of -0.3 V to 0 V (vs. Ag/AgCl), at the frequency determined from the inflection point of a capactive Nyquist plot performed prior to measurement. Continuous redox capacitance measurements were conducted at the half-wave potential of the interface with the same AC frequency as determined from the inflection point of a capacitive Nyquist plot performed prior to measurement. Square-wave voltammetry (SWV) was conducted from -0.3 V to 0 V (vs. Ag/AgCl) with an amplitude of 20 mV, a potential step of 5 mV and a frequency of 50 Hz in 0.1 M PB buffer with a pH of 7.4.

The Au working electrodes were cleaned according to established methods.[2] The Au disc electrodes were first mechanically polished using a slurry of MicroPolish alumina powder (Buehler) of decreasing size: 1.0, 0.3, and 0.05 μm in sequence. The electrodes were sonicated briefly in 1:1 deionized water/ethanol 3 times/each, rinsed with deionized water then immersed in piranha solution (conc. H_2SO_4 / 30% H_2O_2, v/v 3:1) for approximately 15 mins. Following this, they were rinsed throroughly again with deionized water then electrochemically polished, first in 0.5 M KOH (aq) with repeat scans by cyclic voltammetry from - 0.7 to - 1.7 V (vs. Ag/AgCl), then in 0.5 M H_2SO_4 (aq) from - 0.15 V to + 1.35 V (vs. Ag/AgCl) with a scan rate of 0.1 $V \cdot s^{-1}$, both for approximately an hour. The electroactive surface area of the gold disc electrode was estimated by dividing the integral of the gold reduction peak in 0.5 M H_2SO_4 by 420 $\mu C \cdot cm^{-2}$.[3]

SPR kinetic study

SPR gold chips (SIA kit Au, Cytiva) were first rinsed with ethanol, then deionized water several times before submerging the chips in piranha acid (same composition as above) for 10 min. The chips were then rinsed thoroughly with deionized water,

before placing the chips in an aniline solution (1 mL 98 % aniline and 2 mL 50 % phytic acid into 17 mL of deionized water) for chronopotentiometric electrodeposition of PANI, performed at a current density of 10 $\mu A \cdot cm^{-2}$ for 10 min. Antibodies were immobilised onto the PANI-coated chip in situ using glutaraldehyde as a crosslinker, which was achieved by placing the PANI-coated chip into the SPR spectrometer and flushing the film with 0.1 M PB buffer (pH = 7.4) at a flow rate of 30 μL/min at room temperature, followed by an 30-min incubation under flow with 2.5% glutaraldehyde (in PB buffer). Finally, the chips were exposed to a solution of 100 $\mu g \cdot mL^{-1}$ anti-CRP, and left to incubate for 30 min. The antibody coverage was estimated using the standard Biacore working protocol where $c_{\text{surface concentration}}=1.0 \cdot \Delta RU\ pg \cdot mm^{-2}$, where the ΔRU is the difference of response units between the channel injected samples and control channel which injects PB buffer only.[4] A full SPR sensogram was constructed by injecting a series of increasing concentrations of recombinant CRP (from 39.1 $ng \cdot mL^{-1}$ to 10.0 $\mu g \cdot mL^{-1}$) (see Figure **S10**), with the association and dissociation regimes lasting approx. 270 s and 600 s, respectively. The kinetic behaviour of CRP binding was studied by fitting the association regime of each CRP sample injection with Equation **3** to obtain the observed rate constant, k_{obs}, which is defined as $c_{analyte} \cdot k_{on} + k_{off}$. By plotting k_{obs} versus analyte concentration, $c_{analyte}$, the CRP binding rate constant can be estimated at the point at which the slope is equivalent to association rate constant, k_{on}, and the intercept corresponds to the dissociation rate constant, k_{off}.

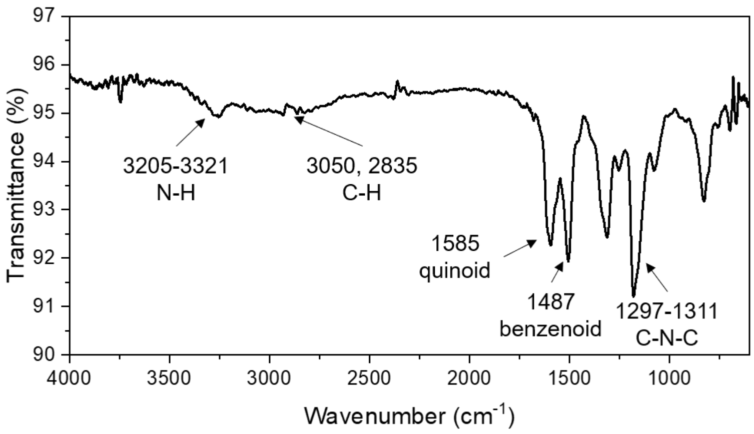

Figure S1. FT-IR spectrum of the PANI-coated gold chip, including a broad band of secondary amine N-H stretching vibrations from 3205 to 3321 cm^{-1}, a sharp peak of quinoid C=C stretching at 1586 cm^{-1} and the benzenoid C=C stretching at 1487 cm^{-1}.

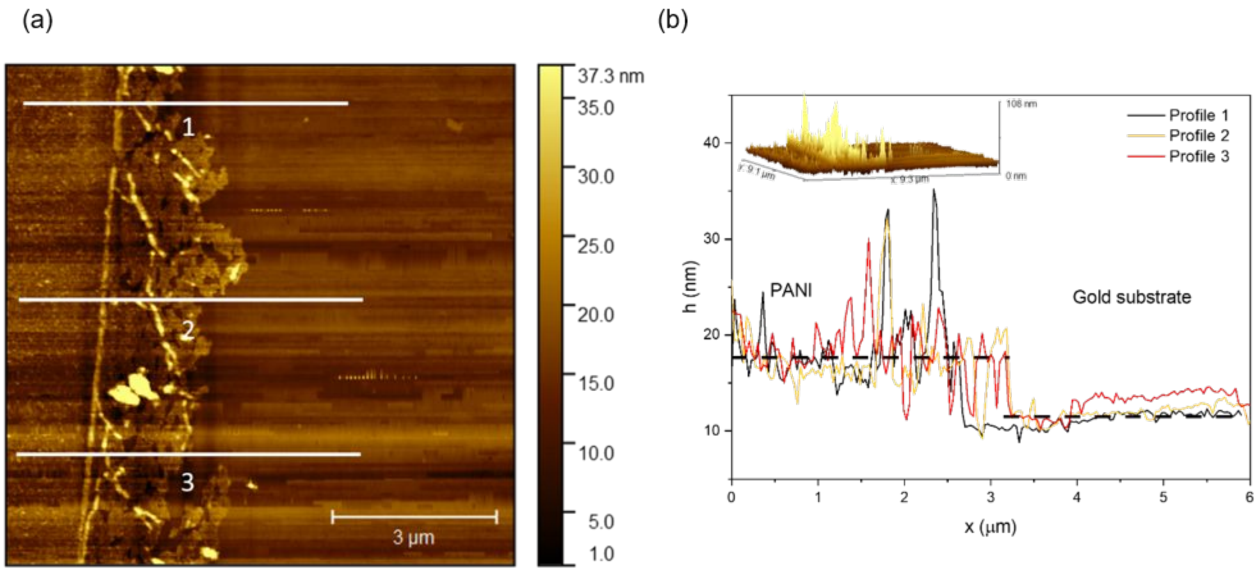

Figure S2. (a) An atomic force microscopy (AFM) image of the PANI interface electrodeposited on gold, where the left side of the bright boundary depicts the PANI film, the middle bright line is the deposit boundary, and the darker side to the right is the unmodified gold substrate. (b) Three cross-sectional line profiles of the PANI interface, taken from the lines depicted in Figure **S2a**; inset: 3D view of the PANI interface shown in Figure **S2a**. The thickness of the PANI interface was estimated by averaging the height difference between the PANI interface and gold substrate across the three profiles. The surface roughness was calculated from the arithmetic average of profile height deviations from the mean line.

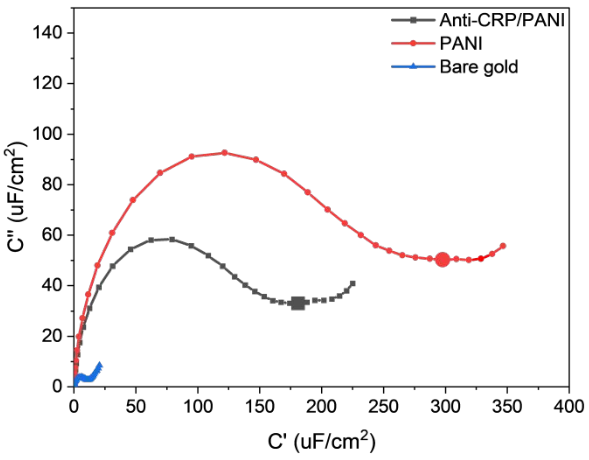

Figure S3. Capacitive Nyquist plots of a bare gold electrode (blue), after modification with PANI film (red), and glutaraldehyde/anti-CRP/PANI (black) interface measured in 0.1 M PB buffer with a pH of 7.4 at a potential of -0.16 V vs. Ag/AgCl. The enlarged labels are the inflection points of the curves, corresponding to the C_r of the interface and the point at which f_r is determined to be applied during continuous measurements.

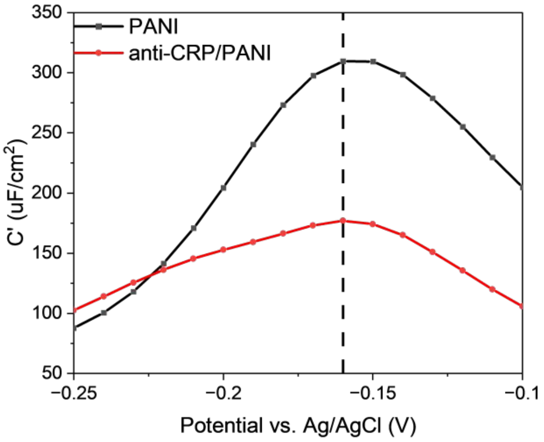

Figure S4. Density of states measurement showing redox capacitance as a function of potential for a PANI film (black line) on a gold disc electrode, and an antibody modified anti-CRP/PANI interface (red line), with an AC frequency selected from the inflection point in capacitive Nyquist plots (see Figure **S3**), measured in 0.1 M PB buffer with a pH of 7.4. The dashed line at -0.16 V vs. Ag/AgCl corresponds to the half-wave potential of the interface.

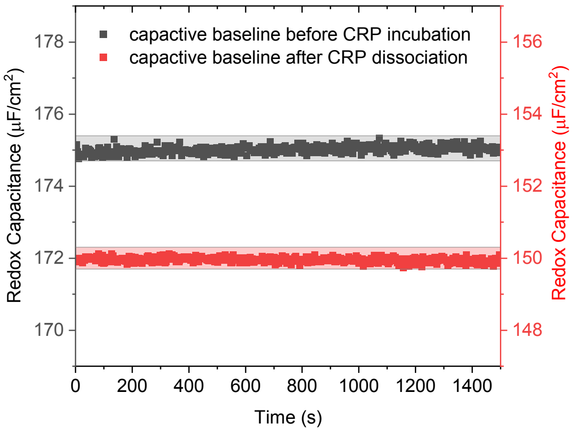

Figure S5. The capacitive baseline stability of anti-CRP/PANI interface before (black) and after 10-min CRP incubation (red), measured in 0.1 M PB buffer with a pH of 7.4. Redox capacitance data was monitored from the inflection points of the capacitive Nyquist plots in Figure **S3**.

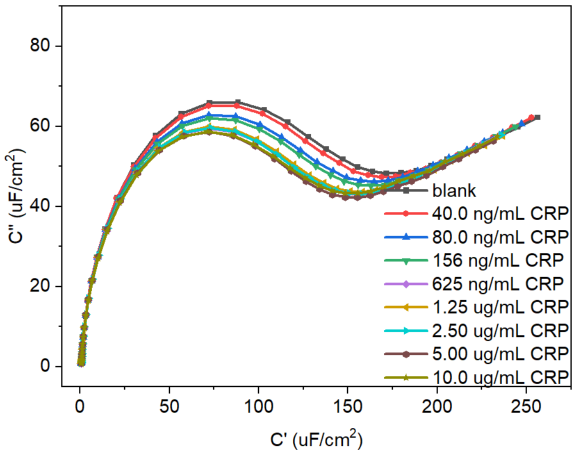

Figure S6. End-point capacitive Nyquist plots of the anti-CRP/PANI interface in reponse to varying concentrations of CRP from 40.0 ng·mL^{-1} (343 pM) to 10.0 μg·mL^{-1} (87.7 nM), at the half-wave potential of PANI/PANI^{+} (-0.16 V), where the redox capacitance, C_r is measured at the inflection point (corresponding to a frequency of 14.25 Hz). Langmuir-Freundlich fitting (R^2 = 0.991) was applied to the resolved binding isotherm to determine a dissociation constant (K_d) of 33 nM.[2]

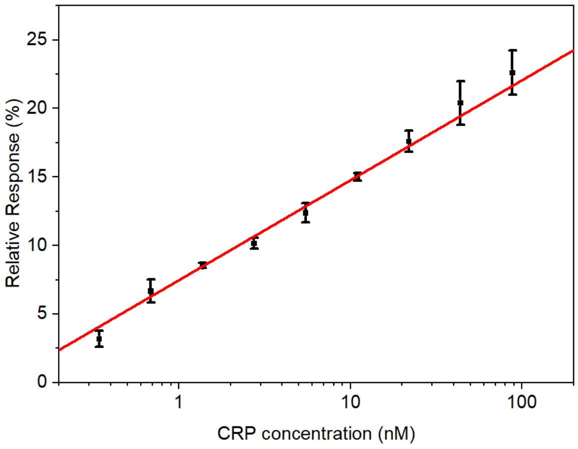

Figure S7. Representative end-point capacitive relative responses to varying CRP concentrations, showing a dynamic range from 80.0 ng·mL^{-1} (651 pM) to 10.0 µg·mL^{-1} (87.7 nM). Error bars represent the standard deviation of three individual electrodes (n = 3).

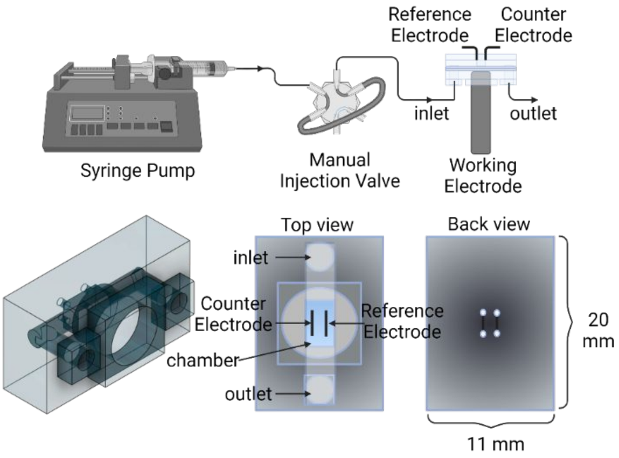

Figure S8. Schematic depiction of the microfluidic continuous flow setup used herein, including a syringe pump connected to a manual injection valve that delivers the sample and reagents to a 3D printed microfluidic cell equipped with a Ag/AgCl wire pseudoreference electrode and Pt wire counter electrode, as well as a 6.4 mm Au disc electrode (Au diameter is 1.6 mm).

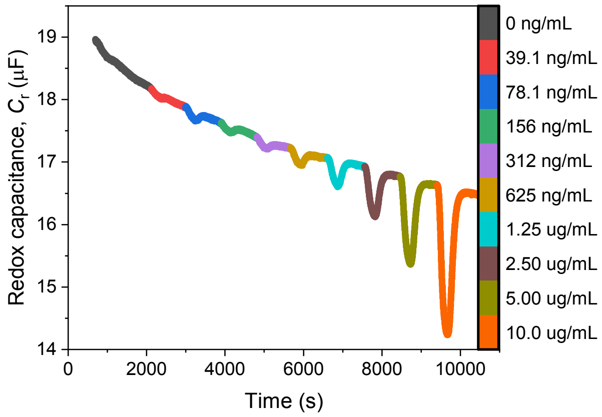

Figure S9. An ECS sensogram of the anti-CRP/PANI interface in response to increasing CRP concentrations, under continuous flow at a flow rate of 25 μL·min^{-1}. The obtained responses were converted into relative responses according to the equation RR/% = (1- $C_{r,t}$)/ $C_{r,0}$ *100, where $C_{r,0}$ is the redox capacitance before sample injection. Further details concerning the method used to perform continuous flow assays can be found in the Experimental section of the manuscript.

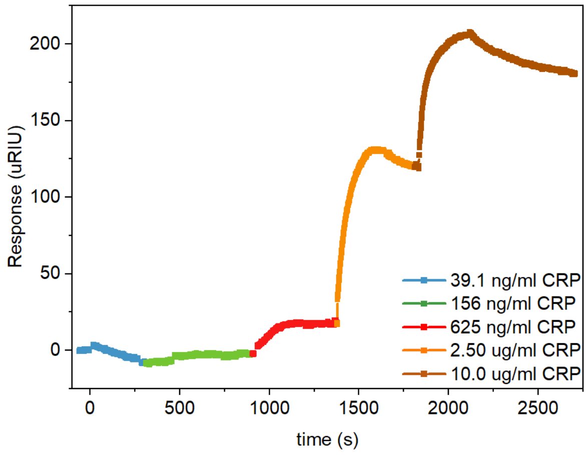

Figure S10. An SPR sensogram of the anti-CRP/PANI interface to increasing CRP concentrations under continuous flow, at a flow rate of 30 μL·min^{-1}. The resolved data was fitted to Equation **3** to obtain the association/dissociation rate constant of CRP binding (shown in Figure **S9**).[2]

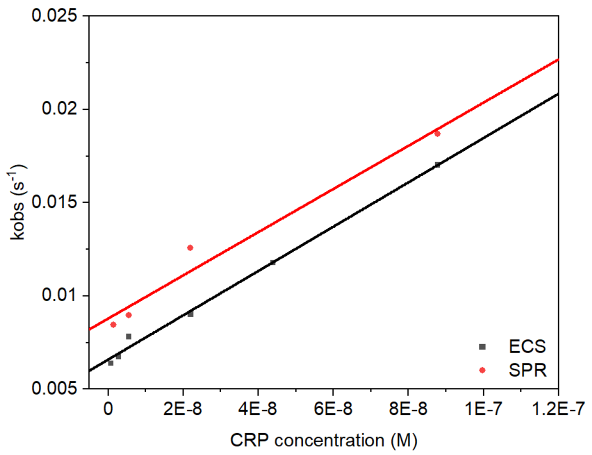

Figure S11. Comparison of the observed rate constant, k_{obs} versus CRP concentration, as determined by continuous-flow ECS (black) and SPR (red) where the respective association rate constants (found from the slope) are k_{on} = 1.19 × 10^5 $M^{-1}s^{-1}$ (ECS) and k_{on} = 1.16 × 10^5 $M^{-1}s^{-1}$ (SPR), and the respective dissociation rate constants (intercept) are k_{off}= 6.58 × 10^{-3} s^{-1} (ECS) and k_{off} = 8.78 × 10^{-3} s^{-1} (SPR).

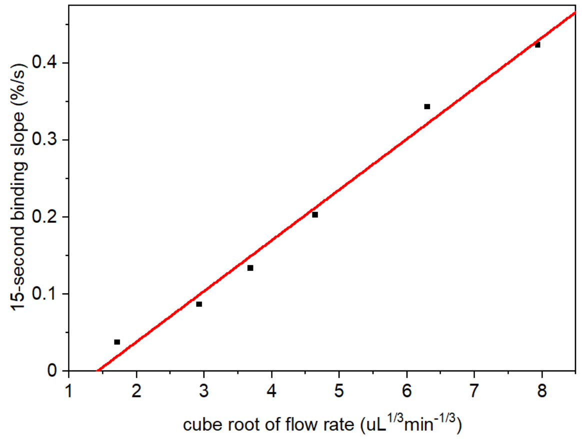

Figure S12. The influence of flow rate (5, 25, 50, 100, 250, 500 μL·min^{-1}) on the ECS signal slope during a 15 s sampling regime, as measured from the response of the anti-CRP/PANI interface to incubation of 10.0 μg·mL^{-1} CRP in PB buffer solution (pH = 7.4).

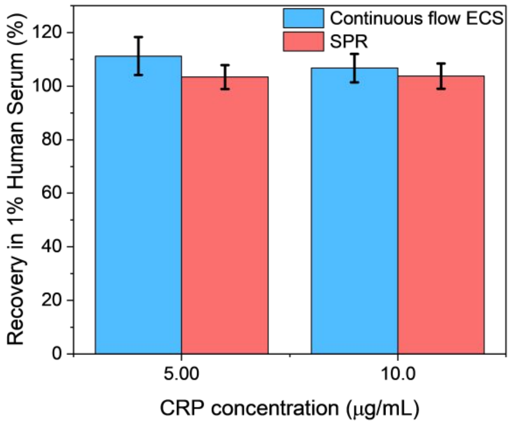

Figure S13. The recovery of an anti-CRP/PANI interface with 5.00 and 10.0 μg·mL^{-1} CRP spiked in 1.0 % human serum, as measured under continuous flow by real-time ECS (blue bars) and SPR (red bars) measurements. Error bars represent the standard deviations of three individual measurements (n = 3).

Time Estimation for CRP binding equilibrium

An accurate estimation of the time taken during each stage of CRP binding (mixing, equilibration, association and dissociation) is essential to determining which region of data should be sampled. i.e. at a given time, does binding reaches equilibrium?

Equation **3,** describing association, is shown as followed,

$$f_b = f_{b,eq}[1 - exp(- k_{obs}t)]\#(3)$$

Where f_b is the fraction of bound target, defined as the number of binding sites over the total number of available binding sites. $f_{b,eq}$ is the fraction bound at equilibrium, defined as $f_{b,eq} = \frac{c}{c + K_d}$, and k_{obs} is the observed rate constant obtained from $k_{obs} = k_{on} c + k_{off}$, where t is the association time.

From Equation **3**, the time required to reach equilibrium can be calculated as,

$$t_{eq} = - \frac{1}{k_{obs}}\ln\left[1 - \frac{f_b}{f_{b,eq}}\right]\#(S1)$$

Using Equation **S1**, the time required to reach CRP binding equilibrium is calculated as follows:

Binding ratio / CRP concentration (nM)	90 % / min	95 %/ min	99 %/ min
0.326	5.1	6.6	10.2
0.651	5.1	6.6	10.1
1.30	5.0	6.5	10.0
2.60	4.9	6.4	9.9
5.21	4.8	6.2	9.5
10.4	4.4	5.8	8.9
20.8	3.9	5.1	7.8
41.7	3.2	4.1	6.4
83.3	2.3	3.0	4.6

Table S1. Time required for specific CRP binding ratio ($f_b/f_{b,\,eq}$), where the association rate constant for CRP binding is 1.10×10^5 $M^{-1}s^{-1}$ while the dissociation rate constant is 7.5×10^{-3}. Results show that in 3-5 mins, 90% of CRP will bind to the surface. In this work, with a flow rate of 25 μL·min^{-1} and sample volume of 100 μL, CRP binding is expected to reach quasi-equilibrium.

Proof of association kinetics by linear approximation (with Taylor's theorem)

The exponential function in Equation **3** can be presented as a Taylor series:

$exp(-x) = 1 - x + \frac{x^2}{2!} - \frac{x^3}{3!} + \cdots \#(S2)$when $x \to 0$,

$$exp(-x) \approx 1 - x \ \#(S3)$$

And the Taylor remainders were defined as:

$$R(-x) = \frac{x^2}{2!} - \frac{x^3}{3!} + \cdots \#(S4)$$

Combining Equation **3** and **S3**, we obtain Equation **S5**:

$f_b \approx f_{b,eq} k_{obs} t = k_{on} \cdot c \cdot t \ \#(S5)$

But since biomarker association rate constant is fixed, we arrive at Equation **S6**, showing that the initial change of signal is proportional to the biomarker concentration:

$$rate = \frac{\partial f_b}{\partial t} = k_{on} \cdot c \ \#(S6)$$

Calculating the relative error associated with different data sampling durations

A linear regression $f_b(t)$ is shown as follows,

$$f_b = kt + b \#(S7)$$

Where k and b are the slope and intercept of the linear regression $f_b(t)$ respectively.

The linear regression $f_b(t)$ must span the data average $[\bar{t}, \overline{f_b}]$. From the association equation (Equation **3**), this affords Equations **S8** and **S9**:

$$\bar{t} = \frac{1}{t_0}\int_0^{t_0} t dt = \frac{t_0}{2} \#(S8)$$

$$\overline{f_b} = \frac{1}{t_0}\int_0^{t_0} f_b dt = \frac{f_{b,eq}\left(k_{obs}t_0 + e^{-k_{obs}t_0} - 1\right)}{k_{obs}t_0} \#(S9)$$

Thus,

$$k = \frac{\sum_{i=1}^{n}(t_i - \bar{t})(f_b - \overline{f_b})}{\sum_{i=1}^{n}(t_i - \bar{t})^2} = \frac{6f_{b,eq}e^{-k_{obs}t_0}\left(k_{obs}t_0e^{k_{obs}t_0} + k_{obs}t_0 - 2e^{k_{obs}t_0} + 2\right)}{k_{obs}^2t_0^3} \#(S10)$$

when $x \rightarrow 0$, the initial rate of association equation is shown as follows,

$$k_0 = f_{b,eq}k_{obs} \#(S11)$$

The relative error of linear fitting is therefore defined by Equation S12:

$$E_r = \frac{|k - k_0|}{k_0} \#(S12)$$

Combining equation **S10-S12**, we obtain Equation **S13**:

$$E_r = \left|\frac{6e^{-k_{obs}t_0}\left(k_{obs}t_0e^{k_{obs}t_0} + k_{obs}t_0 - 2e^{k_{obs}t_0} + 2\right)}{k_{obs}^3t_0^3} - 1\right| \#(S13)$$

As shown in Figure **S14**, the relative error associated with linear fitting to the initial rate increases with longer data sampling windows (Table **S2**). This increasing deviation comes from a slower dissociation rate as surface concentration of the antigen-antibody complex increases. The error is approximately constant below the optimum sampling window time (i.e., < 15 s), but contains insufficient data points to support an accurate determination of the slope. Therefore, the optimum sampling window time is 15 seconds. In the case of 625.0 ng·ml^{-1} CRP assay, the relative error is 5.84 % when fitting to a 15-second sampling window.

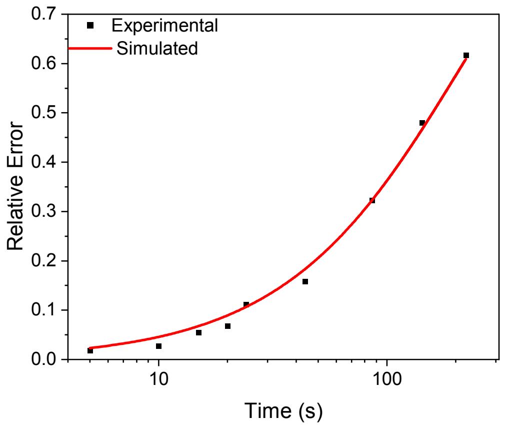

Figure S14. Experimental (black) error of the relative response slope versus the sampling time length in response to 625.0 ng·ml^{-1} CRP binding to the anti-CRP/PANI interface. The red line represents fitting with Equation **S13**, assuming that k_{obs} = 9.38 × 10^{-3} s^{-1}, as determined from SPR measurements, see Figure **S10**.

Time length	15 s	30 s	60 s	120 s
Relative error	5.84%	11.3%	21.0%	40.0%

Table S2. Estimated relative error of initial binding slope obtained from Equation **S13** with a given sampling window in response to 625.0 ng·ml^{-1} CRP binding.

REFERENCE

(1) Bueno, P. R.; Mizzon, G.; Davis, J. J. Capacitance spectroscopy: a versatile approach to resolving the redox density of states and kinetics in redox-active self-assembled monolayers. *J. Phys. Chem. B* **2012**, *116* (30), 8822-8829.

(2) Baradoke, A.; Hein, R.; Li, X.; Davis, J. J. Reagentless redox capacitive assaying of C-reactive protein at a polyaniline interface. *Anal. Chem.* **2020**, *92* (5), 3508-3511.

(3) Trasatti, S.; Petrii, O. Real surface area measurements in electrochemistry. *Pure Appl. Chem.* **1991**, *63* (5), 711-734.

(4) Markey, F. Principles of Surface Plasmon Resonance. In *Real-Time Analysis of Biomolecular Interactions: Applications of BIACORE*, Nagata, K., Handa, H. Eds.; Springer Japan, 2000; pp 13-22.
